# The new application of mifepristone in the relief of adenomyosis-caused dysmenorrhea

**DOI:** 10.7150/ijms.39252

**Published:** 2020-01-14

**Authors:** Xuan Che, Jianzhang Wang, Jiayi He, Xinyue Guo, Tiantian Li, Xinmei Zhang

**Affiliations:** 1 Women's Hospital, School of Medicine, Zhejiang University, Hangzhou, Zhejiang, P.R. China, 310006; 2 Jiaxing Maternity and Child Health Care Hospital, Jiaxing, Zhejiang, P.R. China, 314000

**Keywords:** adenomyosis, dysmenorrhea, mifepristone, inflammation, mast cell, nerve fiber

## Abstract

**Background**: Adenomyosis is a quite common gynecological disorder and above 30% of patients have typical secondary and progressive dysmenorrhea. Current treatments still have many disadvantages and thereby the novel treatment aiming to relieve dysmenorrhea still needs to be further investigated. Mifepristone is a wonderful drug because it is effective, safe and cheap in many diseases including adenomyosis. In this study, we aim to investigate if mifepristone could be used in the treatment of adenomyosis-associated dysmenorrhea.

**Methods**: Human primary endometrial epithelial and stromal cells from adenomyosis patients were isolated and treated with mifepristone. RNA-sequencing was then performed to detect the gene changes of pain-related inflammatory mediators. Meanwhile, the effect of mifepristone on the infiltration and degranulation of mast cells were investigated in adenomyosis lesions. Additionally, the role of mifepristone on the density of nerve fibers was also studied in the ectopic endometrium. At last, to evaluate the therapeutic efficacy of mifepristone on dysmenorrhea of adenomyosis, twenty participants were included and the visual analog scale (VAS) score was assessed and compared before and after treatment with mifepristone.

**Results**: We demonstrated that mifepristone reduced the secretion of IL-6 and TNF-α from endometrial epithelial and stromal cells, restricted the infiltration and degranulation of mast cells in eutopic and ectopic endometrium and decreased the density of nerve fibers by inhibiting the migration capacity of nerve cells in adenomyosis. Meanwhile, we found that mifepristone could significantly relieve dysmenorrhea of adenomyosis.

**Conclusion**: The findings demonstrated that mifepristone could be applied in the treatment of dysmenorrhea for the adenomyosis patients.

## Introduction

Adenomyosis is defined as invasion of endometrial glands and stroma into the myometrium and the prevalence of adenomyosis ranges from 8 to 27% of women in reproductive age 1. Adenomyosis causes many health problems such as dysmenorrhea, hypermenorrhea and subfertility. Above 30% of patients have typical secondary and progressive dysmenorrhea 2. Serious dysmenorrhea can affect the qualities of work, eating and sleep and cause depression, which restricts the daily routine of these patients and has a tremendous impact on their physical and mental health 2, 3. Moreover, adenomyosis is diagnosed in 20-25% of infertile young women undergoing assisted reproductive technologies 4. Severe dysmenorrhea is the primary reason for patients to choose hysterectomy and lost their fertility. Current therapy for adenomyosis-associated pain includes hysterectomy, oral contraceptive drugs and gonadotrophin-releasing hormone (GnRH) agonists. However, the present medical treatment for adenomyosis-related dysmenorrhea is limited for their side effects such as premenopausal symptoms, high relapse rate after medicine withdrawal and high costs 5, 6. Thus, the novel medical treatment aiming to relieve dysmenorrhea should be further investigated.

The exact pathogenic mechanism of adenomyosis-related dysmenorrhea remains unclear, while inflammation and innervation possibly are the key pathogenic factors 7. Inflammatory mediators, including IL-6, TNF-α, IL-1β and IL-10, are involved in inflammatory pathway and contribute to the intense painful symptoms in adenomyosis 8-10. Furthermore, increasing evidence supports that the activated mast cell is regarded as a director of common inflammatory pathways contributing to chronic neuropathic pain and may play a role in pathogenesis of adenomyosis 11-13. Our previous study also showed that the recruitment and degranulation of mast cells play an important role in endometriosis-related dysmenorrhea 14. In addition, recent research found adenomyosis-induced pain resembles neuropathic pain 15. We also proved that the density of nerve fibers in the functional layer endometrium of adenomyosis patients was correlated with dysmenorrhea, suggesting the nerve fibers play an important role in the mechanisms of pain generation in adenomyosis 16. Obviously, drug therapy for adenomyosis can be based on the above-mentioned pathogenesis of adenomyosis.

Mifepristone is the first and one of the most widely used selective progesterone receptor modulators (SPRM) since 1982. Besides mild adverse effect and well tolerance in its long-term clinical application, low price of this drug is another great advantage for the patients because adenomyosis needs the long-term medical therapy 17. In China, the cost of mifepristone is only less than 4 US dollars per month while GnRH-a treatment needs more than 200 US dollars per month in the treatment of endometriosis. Currently, we find that mifepristone has more benefits for human health than what we thought before. Recent studies showed that mifepristone strongly decreased the levels of tumor necrosis factor-α (TNF-α), interleukin-1β (IL-1β) and interleukin-6 (IL-6) of paraquat-induced lung injury in rats 18. Treatment of mifepristone significantly downregulated the expression of neuronal nitric oxide synthase (nNOS) and N-methyl-D-aspartate receptor subunit 2B (NR2B) proteins in a rat model of radicular pain 19. Furthermore, Li et al. reported that addition of mifepristone to depot-medroxyprogesterone acetate (DMPA)-exposed endometrium significantly decreased mast tryptase-positive cells and pointed that mifepristone is associated with inhibiting the activity of mast cells 20. Some studies in China and we also found that mifepristone could be applied in the treatment of adenomyosis. Taken together, theoretically, mifepristone may be a new therapeutic agent for adenomyosis-related pain. However, only a few studies were performed to investigate the role of mifepristone on the dysmenorrhea caused by adenomyosis.

In this study, human primary endometrial epithelial cells and stromal cells from adenomyosis patients were isolated and treated with mifepristone. RNA-sequencing was then performed to detect the gene changes of inflammatory mediators. Meanwhile, we investigated the effects of mifepristone on the infiltration and degranulation of mast cells in adenomyosis. Additionally, we investigated the role and mechanism of mifepristone on the density of nerve fibers in the ectopic endometrium of adenomyosis patients. At last, to further study the therapeutic efficacy of mifepristone on dysmenorrhea of adenomyosis, twenty participants were included and the visual analog scale (VAS) score was assessed and compared before and after treatment with mifepristone. Our study was performed to elucidate the effect of mifepristone on the relief of dysmenorrhea, which will provide a solid foundation for the application of mifepristone in the treatment of adenomyosis patients with dysmenorrhea.

## Materials and Methods

### Isolation and identification of endometrial stromal and epithelial cells of adenomyosis

Isolation of primary endometrial stromal and epithelial cells was performed using a previously reported method 21. Briefly, the tissues were washed with FBS-free medium under aseptic conditions and were minced into 1×1×1 mm^3^ pieces. After the minced tissues were digested with 1 mg/mL collagenase type III at 37°C for 60 min, the endometrial epithelial cells and stromal cells were separated by two sequential filtrations of 200 and 70 μm cell strainer. Endometrial epithelial cells remaining in the cell strainer were collected were cultivated in primary epithelial growth medium (PriCells, Wuhan, China) and endometrial stromal cells were cultivated in Dulbecco's modified Eagle's medium (DMEM)/F12 medium (Thermo Fisher, CA, USA) supplemented with 10% FBS (Sigma-Aldrich, MO, USA). Identification of the isolated endometrial epithelial cells and stromal cells was assessed with anti-cytokeratin and anti-vimentin antibodies by immunohistochemistry as described in our previous study 22.

### Cell lines

The RBL2H3 mast cell line was purchased from Stem Cell Bank, Chinese Academy of Sciences and cultivated in minimum Eagle's medium (Sigma) supplemented with 10% heat-inactivated fetal calf serum (Gibco), 100 U/mL penicillin and 100 μg/mL streptomycin. PC12 nerve cell line, as a neuronal model, was purchased from Chinese Academy of Sciences and cultured in a complete medium consisting of 85% F-12 medium (Sigma), 10% heat-inactivated horse serum (Gibco), and 5% fetal calf serum (Gibco). RBL2H3 Cells and PC12 Cells were treated with or without mifepristone at concentration of 50 μM for 48h in this study.

### The Cell-counting Kit-8 (CCK-8) Assay

To investigate the suitable concentration of mifepristone treatment for the following study, primary endometrial epithelial cells were treated with mifepristone in different concentrations (0, 10, 25, 50, 75, 100 and 200 μM, respectively) for 24h. Then, the viability of above cells was detected by the CCK-8 assay (Biosharp, Beijing, China). CCK-8 reagent was added to each well and cells were incubated at 37°C for 2h in accordance with the manufacturer's instructions. The absorbance at 450 nm (optical density) was detected to calculate the cell viability.

### High-throughput sequencing

Based on the result of CCK-8 assay, the viability of endometrial epithelial cells was significantly decreased when the concentrations of mifepristone treatment were 75 μM. Subsequently, primary endometrial epithelial cells were treated with mifepristone at the concentration of 50 μM for 24 hours. The epithelial cells were isolated from four independent samples. Total RNAs were isolated using Trizol reagent (Life Technologies, Grand Island, USA) and subjected to RNA high-throughput sequencing by The Beijing Genomics Institute (BGI, Shenzhen, China).

### Real-time polymerase chain reaction

Total RNA of each endometrium was extracted using Trizol reagent (Takara, Japan) and reverse transcription was performed using PrimeScript Reverse Transcription reagent kit (Takara, Japan). RT-PCR was performed using SYBR Premix Ex Taq TM Kit (Takara, Japan) with ABI 7500 realtime PCR system (Thermo, MMAS, USA). The primer was designed using Primer 3, and the nucleotide sequences of IL-6 were as follows: sense 5′-CCTCCAGAACAGATTTGAGAGTAGT-3′; and antisense 5′-GGGTCAGGGGTGGTTATTGC-3′; the nucleotide sequences of TNF-A were as follows: sense 5′-CGAGTGACAAGCCTGTAGCC-3′; and antisense 5′-TGAAGAGGACCTGGGAGTAGAT-3′. As an internal control, GAPDH was also amplified and the nucleotide sequence for the primers were as follows: sense 5′-GCCATCAATGACCCCTTCATT-3′ and antisense 5′-TGACGGTGCCATGGAATTT-3.

### Measurement of mast cell degranulation

As our previous study described, RBL2H3 cell degranulation was measured through the release of β-hexosaminidase 14. RBL2H3 cells were seeded in 96-well plates (5 × 10^4^ cells /well) with or without mifepristone treatment at the concentration of 50 μM for 48h. Then stimulated with DNP-BSA at the concentrations of 100 ng/ml (A6661, Sigma, USA) and degranulation was detected by the release of hex according to the protocol 23.

### Enzyme linked immunosorbent assay (ELISA)

The endometrial epithelial cells and stromal cells were treated with mifepristone at different concentrations (0, 50 and 100 μM, respectively) for 48 h and the concentration of IL-6 and TNF-α protein in endometrial epithelial and stromal cells culture supernatant were detected by ELISA kits of interleukin-6 (IL-6) (ELH-IL6-1, RayBiotech, Peachtree Corners, GA, USA) and tumor necrosis factor-α (TNF-α) (ELH-TNFa-1, RayBiotech, Peachtree Corners, GA, USA). ELISA was performed according to manufacturer's instructions.

### Immunohistochemical staining

Adenomyosis eutopic endometrium and corresponding ectopic endometrium were collected during surgery. The diagnosis of adenomyosis was confirmed by imaging or histological examination. Samples were collected in the proliferative phase of the menstrual cycle. Sections were incubated with anti-c-kit antibody (dilution 1:200, ab32363, Abcam, Cambridge, MA, USA) and anti-PGP9.5 antibody (dilution 1:500, Z5116, Dako Cytomation, DenmarkA/S). Immunohistochemical assay was performed as previously described 24. Individual nerve fibers were then counted under high power (× 200) to obtain a nerve count in a defined area. The average nerve count in five hot spots was calculated 25.

### Cell migration assay

Cell migration ability was evaluated by transwell chamber assay using 24-well plates with 8.0-μm pore size membranes (BD Biosciences, CA, USA). To study the effect of mifepristone on the migratory ability of nerve cells, PC12 cells with or without pre-treatment of mifepristone at the concentration 50 μM were added into the upper chamber of the insert in 200 μL of serum-free medium, while the lower chamber contained growth media with 10% FBS. After 24h incubation, cells in the upper chamber were removed with a cotton swab and the migrated cells in the lower chamber were fixed with methanol, stained with crystal violet and counted with a microscope (Olympus, Japan). The PC12 cells that passed through the membrane was defined as migrated cells.

### Patients and clinical evaluation

This study was approved by Ethics Committee of Women's Hospital, School of Medicine, Zhejiang University and registered in Chinese Clinical Trial Registry (1800015514). Twenty cases of adenomyosis patients were included after informed consent. No hormone or similar drugs were used for 6 months before treatment. The patients were treated with mifepristone by oral administration at 5 mg per day. The visual analog scale (VAS) was used to evaluate the degree of dysmenorrhea before and after treatment of mifepristone for 3 months. The left end of the VAS was scored as 0 to represent “no pain” while the right end was scored as 10, representing the “most severe pain imagined” 26. The VAS score was self-assessed by each patient prior to treatment. On the other hand, platelet count in serum of adenomyosis patients was obtained, and they were analyzed in the hematology laboratory of our hospital.

### Statistical Analysis

SPSS program version 19.0 and Graph Pad Prism 5 software were applied for statistical analysis. Data are shown as the mean ± Standard Error of Mean (SEM). P values were determined by the two-tailed Student's t test or Mann-Whitney U test when comparing two groups and by a one-way ANOVA when comparing more than two groups. Statistical difference was considered to be significant at a value of P< 0.05 (*), highly significant at a value of P< 0.01 (**) and extremely significant when P< 0.001(***).

## Results

### Mifepristone reduces the secretion of IL-6 and TNF-α from endometrial epithelial and stromal cells in adenomyosis

**Figure 1:**
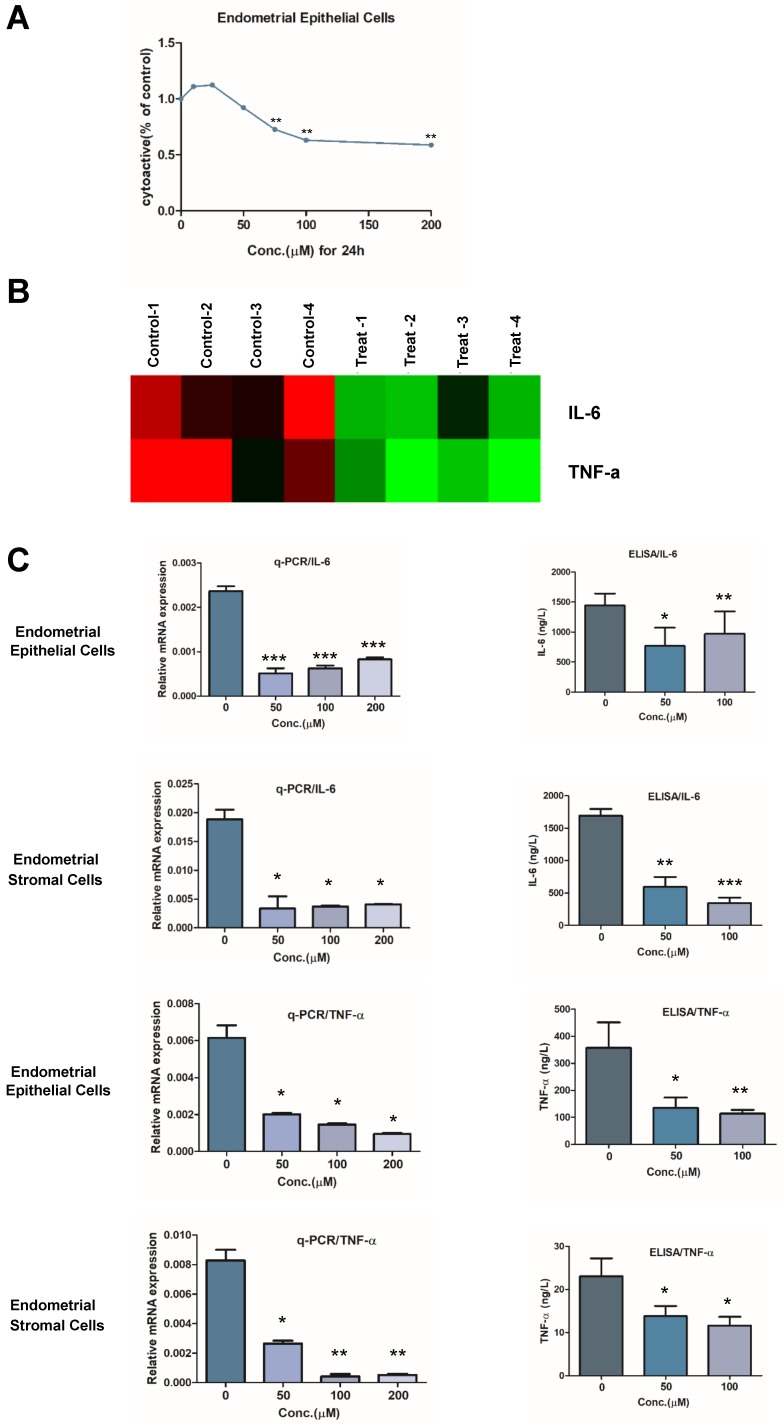
** Mifepristone reduces the secretion of IL-6 and TNF-α from endometrial epithelial and stromal cells in adenomyosis.** (A) Human primary endometrial epithelial cells were treated with mifepristone in different concentrations for 24h, and CCK-8 assay was performed. The viability of endometrial epithelial cells was significantly decreased when treated with mifepristone at 75 μM while there was no significant difference at 50 μM. Concentration at 50 μM was therefore selected for the following RNA-sequencing. (B) Primary endometrial epithelial cells were treated with mifepristone at the concentration of 50 μM and then subjected to next generation sequencing. The endometrial epithelial cells were from four biologically independent samples and the data were shown in quadruplicate. (C) qRT-PCR and ELISA were performed to detect the role of mifepristone on the down-regulations of IL-6 and TNF-α in endometrial epithelial and stromal cells in different concentrations. Data were shown as mean ± SEM. *P<0.05, **P<0.01 and ***P<0.001.

To investigate the potential mechanism of mifepristone relieving dysmenorrhea on the adenomyosis, RNA-sequencing was performed to detect the changes of gene expression in the primary endometrial epithelial cells with or without treatment of mifepristone. Firstly, CCK-8 assay was performed to determine the effective concentrations of mifepristone on the primary endometrial epithelial cells of adenomyosis. Cells were treated with mifepristone at different concentrations (0, 10, 25, 50, 75, 100 and 200 μM, respectively) for 24h. As shown in Fig. 1A, the viability of endometrial epithelial cells was significantly decreased when treated with mifepristone at concentrations above 50 μM. The effective concentration of mifepristone applied in this study was similar to that used in treatments of kinds of cancers 27. Based on the result of CCK-8 assay, the endometrial epithelial cells were treated with mifepristone at the concentration of 50 μM for 24 hours (n=4) and gene expression was examined by RNA-sequencing. Fig.1B showed that mifepristone significantly down-regulated the expressions of IL-6 and TNF-A in endometrial epithelial cells when compared to controls, which are the important pro-inflammatory chemokines closely correlated with dysmenorrhea.

Then, the down-regulations of IL-6 and TNF-α in the mifepristone-treated group were further confirmed by qRT-PCR and ELISA not only in primary endometrial epithelial cells but also in stromal cells. The mRNA expression of IL-6 and TNF-A was decreased in both endometrial epithelial cells and stromal cells when treated with mifepristone treatment in a dose-dependent manner. Subsequently, ELISA assay was conducted to detect the concentrations of IL-6 and TNF-α in cell culture supernatants of endometrial epithelial cells and stromal cells with and without mifepristone treatment. Endometrial epithelial cells and stromal cells were treated with mifepristone at different concentrations (0, 50 and 100 μM) for 48h. We found that the concentrations of IL-6 and TNF-α in cell culture supernatants were significantly decreased in both endometrial epithelial and stromal cells when treated with mifepristone in a dose-dependent manner (Fig. 1C). These results suggested that mifepristone reduces the secretion of IL-6 and TNF-α from endometrial epithelial and stromal cells in adenomyosis and therefore may have an effect on the relief of pain for the adenomyosis patients.

### Mifepristone inhibits the infiltration and activity of degranulation of mast cells in adenomyosis

As well known, mast cells mediate neurogenic inflammation and pain 28. The activated and degranulating mast cells may exert indirect effects on the development of neuropathic pain 29.

**Figure 2:**
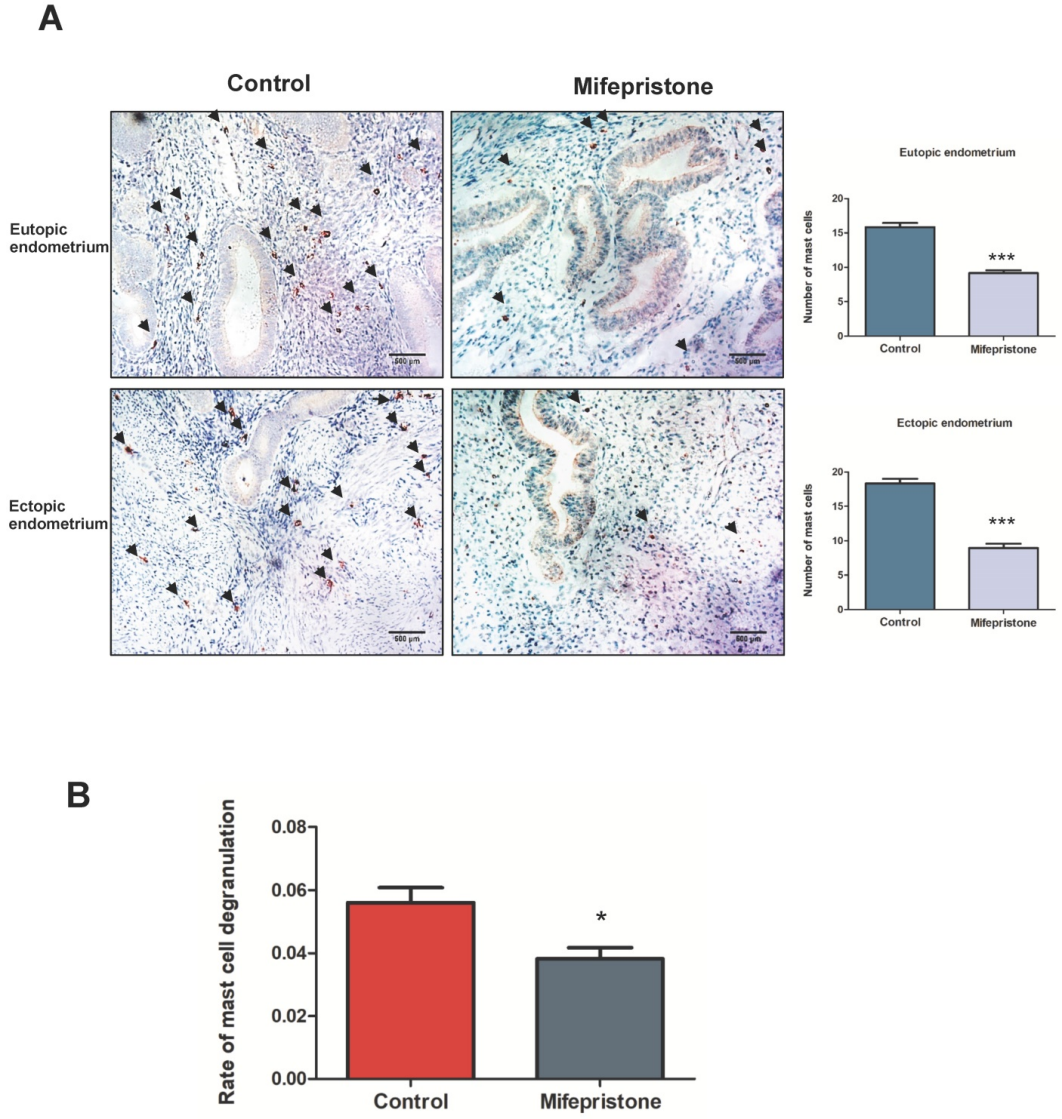
** Mifepristone decreased the number of mast cells in both eutopic and ectopic endometriums and also inhibited the activity of degranulation.** (A) Immunohistochemical staining for c-kit was examined in the eutopic and ectopic endometriums with or without mifepristone treatment. The black arrow indicates the mast cells. Scale bars = 500 μm. Image was captured at 200× magnification. (B) Rate of active degranulation in RBL2H3 mast cells was examined after treatment of 50 μM of mifepristone for 48h. Data were expressed as mean ± SEM. Statistical difference was determined by Mann-Whitney U test. *P<0.05, **P<0.01 and ***P<0.001.

To study the effect of mifepristone on the mast cell-infiltration in adenomyosis, immunohistochemistry was conducted to detect the number of mast cells by staining with c-kit in eutopic and ectopic endometriums of adenomyosis. We observed the numbers of mast cells were significantly decreased in both eutopic and ectopic endometriums after mifepristone treatment (P < 0.001; Fig. 2A). To further determine whether mifepristone has effect on the activity of degranulation of mast cells. RBL2H3 mast cells were treated with mifepristone at concentration of 50 μM for 48h. The rate of degranulation of RBL2H3 cells treated with mifepristone was significantly decreased when compared to mifepristone-untreated group (p<0.05; Fig. 2B). The above results revealed that mifepristone inhibits the infiltration and the activity of degranulation of mast cells in both eutopic and ectopic endometriums of adenomyosis.

### Mifepristone decreases the density of nerve fibers in both eutopic and ectopic endometriums of adenomyosis

**Figure 3:**
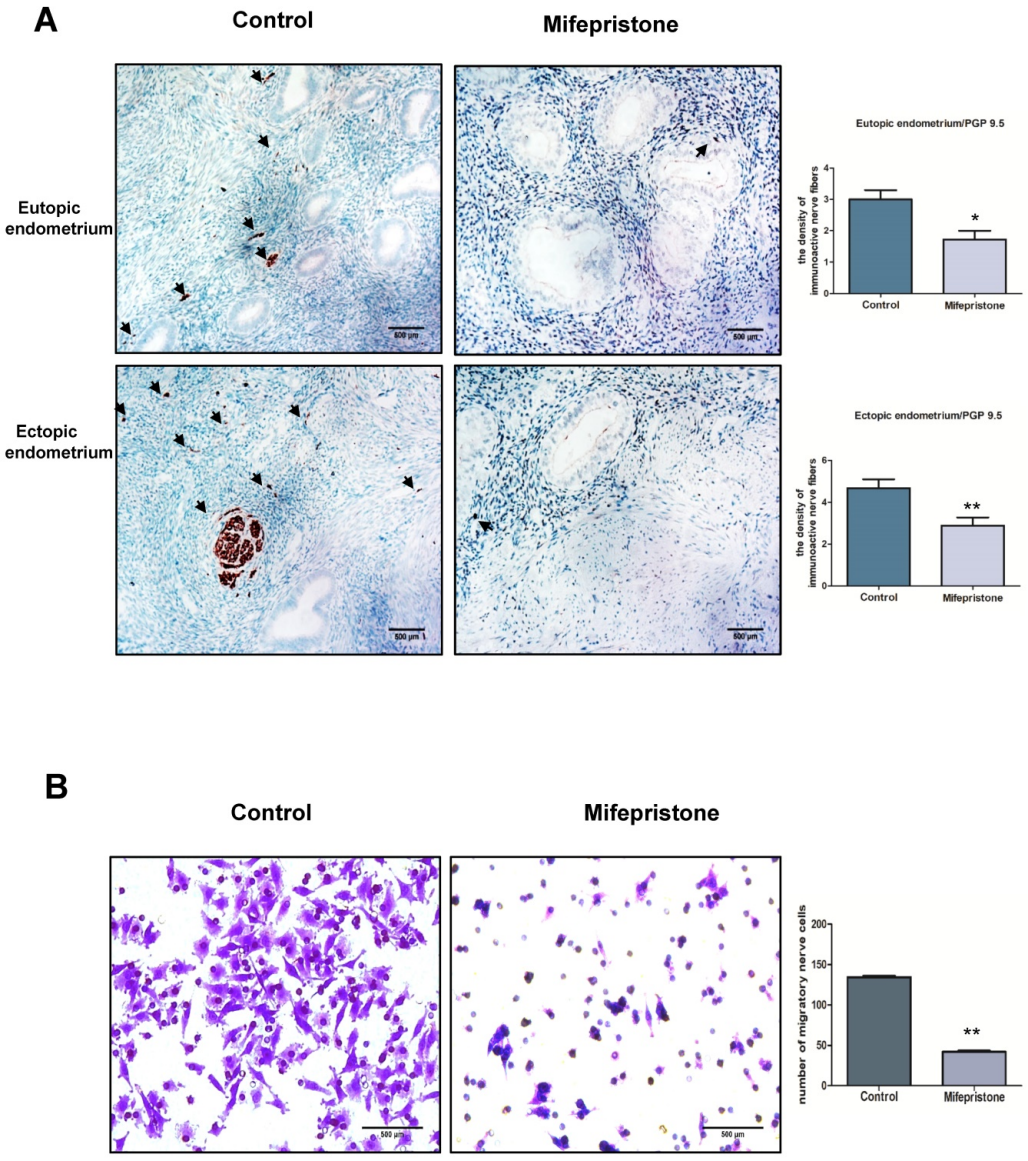
** Mifepristone decreases the density of nerve fibers by inhibiting the migratory capacity of nerve cells in adenomyosis.** (A) Nerve fibers were stained by immunohistochemical staining using PGP9.5 antibody in both eutopic and ectopic endometriums with or without mifepristone treatment. Scale bars = 500 μM. Image was captured at 200× magnification. (B) Phase-contract images of migrated PC12 nerve cells on the bottom of transwell insert membrane with or without treatment of mifepristone. Number of migrated PC12 nerve cells on the bottom of PET membrane was counted as indicated conditions. Data were expressed as mean ± SEM. *P<0.05, **P<0.01 and ***P<0.001.

It is known that afferent sensory fibers are critical for the conduction of adenomyosis-caused pain. Our previous study also found that dysmenorrhea was positively correlated with the density of PGP 9.5-immunoactive nerve fibers in the basal layer of the endometrium and myometrium 30. To investigate the effect of mifepristone on the innervation in adenomyosis, immunohistochemistry was conducted to detect PGP 9.5-immunoactive nerve fibers in endometrium and myometrium tissue with and without mifepristone treatment. Fig. 3A showed that the density of PGP 9.5-immunoactive nerve fibers in mifepristone-treated adenomyosis group was significantly decreased in both eutopic and ectopic endometrium when compared to mifepristone-untreated group, especially in ectopic endometrium of adenomyosis. The findings suggested that mifepristone reduces the density of nerve fibers in adenomyosis, which may play an important role in relieving adenomyosis-caused pain.

### Mifepristone decreases the density of nerve fibers by inhibiting the migration capacity of nerve cells in adenomyosis

To investigate the potential mechanism of how mifepristone decreased the density of nerve fibers in adenomyosis, migration assay was performed to detect the effect of mifepristone on the migratory capacity of PC12 nerve cells in adenomyosis. As shown in Fig. 3B, the number of migratory PC12 cells was significantly decreased in mifepristone-treated group when compared to untreated group in a dose-dependent manner (p<0.000), demonstrating that the migratory ability of nerve cells was significantly restricted after treatment with mifepristone. The above data indicated mifepristone decreases the density of nerve fibers by inhibiting the migratory capacity of nerve cells in adenomyosis.

### Mifepristone significantly relieves dysmenorrhea in adenomyosis patients

**Figure 4:**
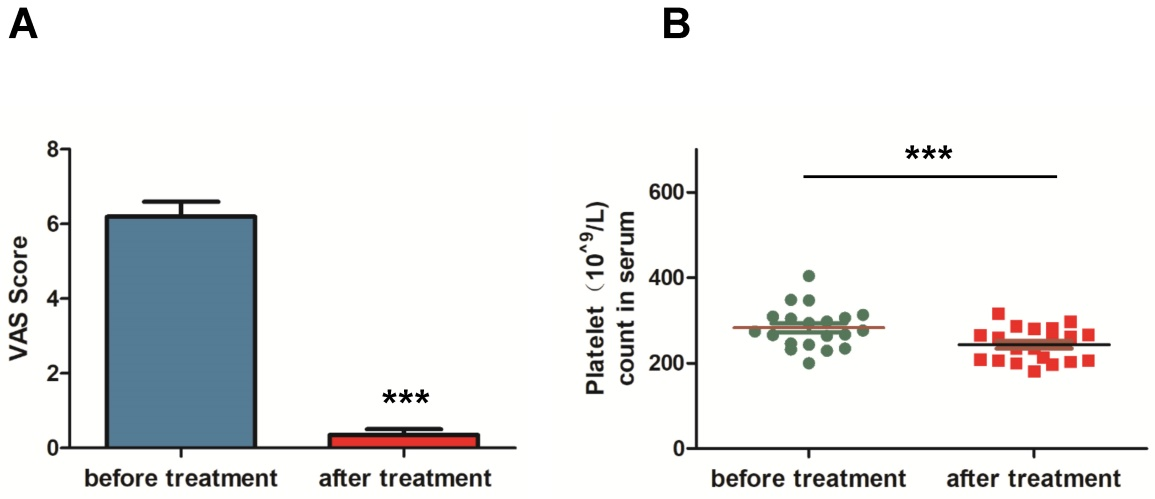
** Mifepristone significantly relieved dysmenorrhea in adenomyosis patients.** (A) The visual analog scale (VAS) score was applied for the pain assessment of dysmenorrhea in the included patients. The VAS score was significantly decreased after mifepristone treatment for the adenomyosis patients. (B) The platelet count in serum of adenomyosis patient was measured before and after three-month treatment with mifepristone. Data were expressed as mean ± SEM. *P<0.05, **P<0.01 and ***P<0.001.

To study the therapeutic efficacy of mifepristone on dysmenorrhea of adenomyosis patients, the VAS score was applied for pain assessment of dysmenorrhea. The VAS score was assessed and compared for the same patient before and after treatment with mifepristone. As shown in Fig. 4A, mifepristone treatment significantly decreased the VAS score of dysmenorrhea for adenomyosis patients when compared to pre-treatment. In addition, it is known that the platelet count in serum is an important marker for the development of adenomyosis and closely associated with dysmenorrhea symptoms. The platelet count in serum before treatment was 282.66 ± 10.84 10^9/L while the mean concentration was 242.95 ± 8.80 10^^9^/L (Fig.4B), indicating that the platelet count in serum were significantly reduced after mifepristone treatment. Therefore, we concluded that mifepristone could relieve dysmenorrhea symptoms for adenomyosis patients.

## Discussion

Dysmenorrhea is a common symptom in adenomyosis and is one of the main reasons that women seek medical treatment. Although medical therapies such as GnRH-a, medroxyprogesterone acetate (MPA) and danazol have shown certain clinical effects for relieving adenomyosis-related dysmenorrhea, the potential side effects compromise those clinical applications. Afferent sensory fibers and proinflammatory mediators are correlated with adenomyosis pain, which can be considered an inflammatory neuropathic pain. Recent studies showed that mifepristone may play important roles in the development of neuropathic pain diseases. However, the evidence for guiding clinical use of mifepristone treatment is insufficient in adenomyosis. The present study will elucidate the feasibility of this old drug for new use in adenomyosis.

Inflammation is a major biological determinant in the pathogenesis of adenomyosis and proinflammatory/inflammatory cytokines act as chemical neurotransmitters to stimulate uterine contraction and cause dysmenorrhea 31. In the present study, we found that mifepristone reduces the secretion of IL-6 and TNF-α from endometrial epithelial and stromal cells in adenomyosis. Similar to MPA and danazol treatment of adenomyosis 32, 33, mifepristone treatment inhibited the secretion of IL-6 in endometrial epithelial and stromal cells of adenomyosis in our experiments. Yang et al. reported that MPA and danazol have no effect on the suppression of TNF-α by endometrial and stromal cells in adenomyosis 34 while our data showed mifepristone significantly decreased the mRNA and protein expression of TNF-α in both endometrial epithelial and stromal cells of adenomyosis. Recent reports pointed that selective progesterone receptor modulators may be possibly more effectively than progestins in relieving adenomyosis-associated pain, but the underlying mechanism was still unclear 35, 36. Our findings showed that mifepristone significantly decreased the expressions of IL-6 and TNF-α in both endometrial epithelial and stromal cells of adenomyosis, which may be the reason that mifepristone is more effectively than progestins in the relief of adenomyosis-associated pain.

Increasing evidence supports that activated and degranulating mast cells play an important role in the development of pain, hyperalgesia and dysmenorrhea 37, 38. Our previous study also demonstrate that the activity and degranulation of mast cells play an important role in endometriosis-related dysmenorrhea 14. Moreover, it is reported that mast cells contribute to the development of inflammation in adenomyosis 10. Therefore, those drugs that can inhibit mast cell-activation and suppress mast cell-degranulation may be used as effective therapeutic agents for adenomyosis. As we expected, our study showed that the infiltration of mast cells was significantly decreased in both eutopic and ectopic endometriums after mifepristone treatment. Moreover, the rate of degranulation of mast cells treated with mifepristone were decreased when compared to mifepristone-untreated group. Hence, we concluded that mifepristone relieved the dysmenorrhea symptom of adenomyosis patients through inhibiting the infiltration and the activity of degranulation of mast cells in eutopic and ectopic endometriums.

It is well known that pain is mediated by sensory nerves. Afferent sensory fibers and proinflammatory mediators are correlated with adenomyosis pain. Our previous study found that the distribution of nerve fibers in the ectopic endometrium play an important role on the pain symptoms in both endometriosis and adenomyosis 16. The present study found that mifepristone decreases the density of nerve fibers in both eutopic and ectopic endometriums of adenomyosis. Furthermore, Transwell assay was then performed to confirm that mifepristone decreased the migration of nerve cells in a dose-dependent manner. Taken together, our data suggested that the relief of adenomyosis-associated dysmenorrhea by mifepristone is related to the decrease of density of nerve fibers by inhibiting the migration capacity of nerve cells in adenomyosis.

At last, the efficacy of mifepristone treatment in adenomyosis was further confirmed by comparing the pain assessment of dysmenorrhea in the same adenomyosis patient before and after mifepristone treatment. We concluded that mifepristone effectively relieved dysmenorrhea symptoms for adenomyosis patients. Furthermore, it is reported that platelets played an important role in the development of adenomyosis and anti-platelet treatment could reduce uterine hyperactivity and improve generalized hyperalgesia 39. Our data showed that mifepristone significantly decreased platelet count in serum of the adenomyosis patients. Therefore, the clinical results further proved that mifepristone was efficient in the treatment of adenomyosis-associated dysmenorrhea and the effect of treatment in adenomyosis is similar to endometriosis 40, 41.

## Conclusion

We firstly demonstrated that mifepristone reduced the secretion of IL-6 and TNF-α from endometrial epithelial and stromal cells, restricted the infiltration and degranulation of mast cells in eutopic and ectopic endometrium and decreased the density of nerve fibers by inhibiting the migratory capacity of nerve cells in adenomyosis. Meanwhile, we found that mifepristone could significantly relieve adenomyosis-associated dysmenorrhea. The findings demonstrated that mifepristone could be applied in the treatment of dysmenorrhea for the adenomyosis patients.
